# Ovarian features in white-tailed deer (*Odocoileus virginianus*) fawns and does

**DOI:** 10.1371/journal.pone.0177357

**Published:** 2017-05-25

**Authors:** G. D. A. Gastal, A. Hamilton, B. G. Alves, S. G. S. de Tarso, J. M. Feugang, W. J. Banz, G. A. Apgar, C. K. Nielsen, E. L. Gastal

**Affiliations:** 1 Department of Animal Science, Food and Nutrition, Southern Illinois University, Carbondale, Illinois, United States of America; 2 Department of Animal and Dairy Sciences, Mississippi State University, Mississippi State, MS, United States of America; 3 Cooperative Wildlife Research Laboratory, Department of Forestry, Southern Illinois University, Carbondale, Illinois, United States of America; Faculty of Animal Sciences and Food Engineering, University of São Paulo, BRAZIL

## Abstract

The knowledge about ovarian reserve is essential to determine the reproductive potential and to improve the methods of fertility control for overpopulated species, such as white-tailed deer (*Odocoileus virginianus*). The goal of this study was to evaluate the effect of age on the female reproductive tract of white-tailed deer, focusing on ovarian features. Genital tracts from 8 prepubertal and 10 pubertal females were used to characterize the preantral follicle population and density, morphology, distribution of follicular classes; stromal cell density; and apoptosis in the ovary. In addition, uterus and ovary weights and dimensions were recorded; and the number and the size of antral follicles and corpus luteum in the ovary were quantified. Overall, fawns had a greater (P < 0.05) preantral follicle population, percentage of normal follicles, and preantral follicle density than does. The mean stromal cell density in ovaries of fawns and does differed among animals but not between age groups. The apoptotic signaling did not differ (P > 0.05) between the ovaries of fawns and does. However, apoptotic ovarian cells negatively (P < 0.001) affected the preantral follicle morphology and density, and conversely, a positive correlation was observed with stromal cell density. As expected, the uteri and ovaries were larger (P < 0.002) and heavier (P < 0.001) in does than in fawns. In conclusion, this study has shown, for the first time, the preantral follicle population and distribution of classes, rate of morphologically normal follicles, and density of preantral follicles and stromal cells in white- tailed deer. Therefore, the findings herein described lead to a better understanding of the white-tailed deer ovarian biology, facilitating the development of new methods of fertility control.

## Introduction

In North America, white-tailed deer (Odocoileus virginianus) are one of the most predominant herbivore species of wildlife. In the late 19^th^ century, this species was considered endangered but has been now considered as overabundant **[1]**. More than any other wildlife species, white-tailed deer have caused a variety of damages, such as: crop loss, automobile and aviation collisions, disease transmission, environmental degradation, and destruction of ornamental plantings, as previously reported **[2, 3]**.

The prevailing abundance of deer has provided the hunting community several opportunities for jobs, food, and sports. Although hunting is facilitating as a method of controlling the deer population, lethal methods are not adequate for effective management in several regions, such as in areas of prohibit hunting, for example in urban areas, national parks and other types of conservation reserves **[4]**. Therefore, wildlife scientists and professionals are developing various non-lethal methods of population control including contraceptive treatments **[5–8]**. However, because of the lack of information on ovarian function of white-tailed deer, more studies are needed to understand the reproductive physiological events to improve the efficiency of contraception and fertility methods.

The white-tailed deer is characterized by a great reproductive fertility, exhibiting multiple parturitions, early sexual maturation (<1 year), and short breeding life (<10 years) **[9]**. In general, the breeding season for white-tailed deer ranges from November to January (Northeast hemisphere). Known reproductive characteristics of mature white-tailed deer **[10]** include: ovulation rate (range, 1 to 3), pregnancy rate (97 to 100%), fecundity (1.6 to 2.0), litter size (1 to 3 per deer), twin rate (>65%), and gestation length (202 days). Although knowledge of ovarian function and follicle population is necessary for implementation of reliable contraceptive methods to control fertility, ovarian reserve of preantral follicles and follicular density have not, to our knowledge, been studied in white-tailed deer. Conversely, studies on preantral follicle population have been conducted for several species, including: laboratory animals (rodents: **[11]**; rabbits: **[12]**), livestock (sheep: **[13]**; goats: **[14]**; horse: **[15]**; cattle: **[16]**), wildlife (macaques: **[17]**; elephants: **[18]**), and humans **[19, 20]**. Furthermore, studies have shown that ovarian reserve reduces as the female ages **[21–23]**. Although the process of germ cell depletion is still not fully understood, one of the main causes seems to be the oxidative stress **[24–26]**.

Although the final goals to use the information obtained from reproductive studies vary among the human biomedical, livestock production, and wildlife management communities, the fundamental tools remain largely the same, and the breadth of communities involved can each benefit from sharing their knowledge and techniques **[27]**. Therefore, the scientific community has also been searching for approaches to be used for fertility preservation studies in endangered mammalian species (e.g. cervidae; **[27, 28]**). Although several wildlife species are, in general, poorly studied in the field of reproductive physiology and gamete preservation, some species have been used more recently as animal models in other fields of research **[29–33]**. Thus, with more applied-specific investigations, white-tailed deer may become a suitable animal model for fertility studies of endangered mammalian species. Therefore, the use of cervidae species as an experimental model may contribute to improve the safeness and effectiveness of non-lethal contraceptive methods to control fertility.

The goal of this study was to evaluate the effect of age on the female reproductive tract features of white-tailed deer fawns and does to: (i) characterize preantral follicle population and density, morphology, and distribution of follicular classes in the ovary; (ii) quantify density of ovarian stromal cells; (iii) evaluate apoptosis in the ovary; (iv) describe macroscopic dimensions and weight of uteri and ovaries; and (v) quantify macroscopic antral follicles, and corpora lutea and albicans.

## Materials and methods

### Female reproductive tracts

Female white-tailed deer genital tracts were harvested during two hunting/reproductive seasons from November to December (fall 2013, n = 13 animals; and fall 2014, n = 5 animals) for macroscopic and microscopic evaluations. All reproductive tracts were obtained from deer harvested in the Southern Illinois region and the Crab Orchard National Wildlife Refuge, Marion, IL, USA (special use permit #14–033). The female deer were categorized into fawns (<1 year of age and prepubertal animals, n = 8), and does (range, 1 to 5 years of age, pubertal, cycling, and nonpregnant animals, n = 10) groups. Age was based on tooth development **[34]** and puberty was based on the presence of corpus luteum and/or corpus albicans in the ovaries.

### Macroscopic evaluations

After recovery, the reproductive tract was dissected free of extraneous tissue, considering the cervix, uterus, oviducts, and ovaries for measurements. The uterus and ovary weights were obtained using a scale in gram (g) units and measurements were taken using a digital caliper in millimeter (mm) units. Briefly, the uterine body length was measured from post-cervix to the uterine bifurcation, and each uterine horn was measured from the uterine bifurcation to the uterotubal-junction. Ovaries were weighted independently and separated from the uterus and oviducts. Measurements of length, height, and width were taken from the ovaries and the volume calculated using the ellipsoid formula (volume = length x height x width x 0.523) **[35]**. Antral follicles and corpora lutea larger than 1 mm were counted, and measured longitudinally through the outer borders of the follicle wall and luteal tissue, respectively. The macroscopic end points consisted of: (a) uterus weight, body length, and horn length; (b) ovary weight, length, height, and width; and (c) presence of ovarian structures (antral follicles, corpus luteum and corpus albicans).

### Histological processing

Ovaries were cut in halves, placed in a 10% neutral-buffered formalin fixative solution for 12 hours at room temperature, and then stored in alcohol (70%) solution in refrigeration (4°C) until histological preparation. Following preparation for histology, all ovaries were embedded in paraffin wax and totally cut into serial sections (7 μm, ≈178 sections per each ovary). Histological sections were mounted on slides and stained with Periodic acid-Schiff (PAS) and hematoxylin. Three slides of each animal were not stained and, therefore, separated for the immunohistochemistry assay.

### Microscopic evaluations

#### Preantral follicle morphology

The histological sections were analyzed using light microscopy (Nikon E200, Tokyo, Japan) at magnification X400 and an image capture system (Leica Imaging Software, Wetzlar, Germany) to determine the total number of preantral follicles in each ovarian section, as well as to classify follicles according to class and morphology, and measure the diameter of follicles and oocyte nuclei. Follicles were classified as normal when the oocyte nucleus and an intact oocyte were present surrounded by granulosa cells that were well organized in one or more layers. Degenerated follicles (abnormal) were defined as those with a retracted cytoplasm or disorganized granulosa cell layers detached from the basement membrane and an oocyte with pyknotic nucleus **[36]**. Moreover, preantral follicles were quantified and classified according to stage of development, i.e., primordial (oocyte surrounded by one layer of flattened granulosa cells with ellipsoidal shape), transitional (one layer of flattened and cuboidal granulosa cells with ellipsoidal shape), primary (one layer of cuboidal granulosa cells with spherical shape around the oocyte), and secondary (oocyte with zona pellucida surrounded by two or more layers of cuboidal granulosa cells; **[37]**). A single operator performed all evaluations and measurements. The microscopic end points consisted of: (a) preantral follicle morphology, classes distribution, density, and population; (b) stromal cell density; and (c) DNA degradation in the ovarian tissue.

#### Preantral follicle measurements and population

Follicle dimensions were measured using a stereoscopic microscope with an ocular micrometer. Follicle diameter was taken from follicles with an intact oocyte (total = 1131, ≈31 preantral follicles /ovary/animal), granulosa cell layer, and basement membrane by the average of two perpendicular measurements from the outer layer of granulosa cells **[36]**. To determine preantral follicle population the nucleus of the oocyte was used as a marker, according to the correction factor previously described **[38]** using the following formula: N_t_ = (N_o_ x S_t_ x t_s_) ⁄ (S_o_ x d_o_), where N_t_ = total calculated number of follicles of one class; N_o_ = number of follicles observed in the ovary; S_t_ = total number of sections in the ovary; t_s_ = thickness of the section (μm); S_o_ = total number of sections observed; and d_o_ = mean diameter of the oocyte nucleus of each follicle class.

#### Preantral follicle density

All slides were scanned and the perimeter of digital images from histological sections was delimited with a photo editing program (Adobe Photoshop CS4, San Jose, USA) after a scale calibration, and the area’s measurement (cm^2^) was recorded. Follicle density was determined by the following formula: follicle density = number of preantral follicles/area of the ovarian section (cm^2^) as previously reported **[23]**.

#### Stromal cell density

Ovarian stromal cell density was evaluated as described previously **[39]**, with some modifications. Briefly, a total of 10% of histological sections from each ovary were analyzed. Five random fields (50 × 50 μm = 2,500 μm^2^) in the cortical area were selected and the stromal cell nuclei were counted to calculate the mean of ovarian stromal cell density.

#### TUNEL assay

Following deparaffinization and rehydration, two histological sections of each ovary from all animals were stained for TUNEL assay using a commercial kit (In Situ Cell Death detection kit, Promega, Madison, WI, USA) according to the manufacturer’s instructions. All tissue sections were examined, along with the number of TUNEL positive cells; the presence of brown coloration in a cell indicated positive staining and apoptosis **[40]**. The proportion of apoptotic cells was measured using ImageJ (version 1.50f) software. Five random fields of each histological section were used to determine the TUNEL positive ovarian cells. The integrated density of staining was calculated according to the following formula: integrated density = total pixel intensity in the selected region / unit area (arbitrary unit, AU). Positive and negative controls were included in every evaluation, according to the manufacturer’s recommendations.

### Statistical analyses

All statistical analyses were performed using R statistical software version 3.0.2 (R Foundation for Statistical Computing, Vienna, Austria). Data for end points that were not normally distributed were transformed to natural logarithms or square root before any statistical analyses. Effects of age (fawns and does) and ovary side (left and right) on the number of preantral follicles were evaluated by two-way ANOVA. The variables without normal distribution (preantral follicle and stromal cell densities, and preantral follicle morphology) were compared by Kruskal–Wallis and Mann-Whitney U test. The variables uterus weight, body length, and horn length; ovary weight, length, height, and width; and ovarian structures (antral follicles, corpus luteum, and corpus albicans) were evaluated by Student unpaired t-test to compare mean values between fawns and does. Spearman’s rank correlation analysis was performed to evaluate the correlation between area of ovarian structures and weight and/or volume. Pearson’s correlation analysis was used to examine the relationships between preantral follicle morphology and density and stromal cell density with apoptotic cells. A simple linear regression analysis was conducted between TUNEL positive cells and preantral follicle morphology. Data are presented as the mean ± S.E.M., unless otherwise stated. A probability of P < 0.05 indicated that a difference was significant, and P > 0.05 and ≤ 0.1 indicated that a difference approached significance.

## Results

### Uterus and ovary measurements

**Fig 1:**
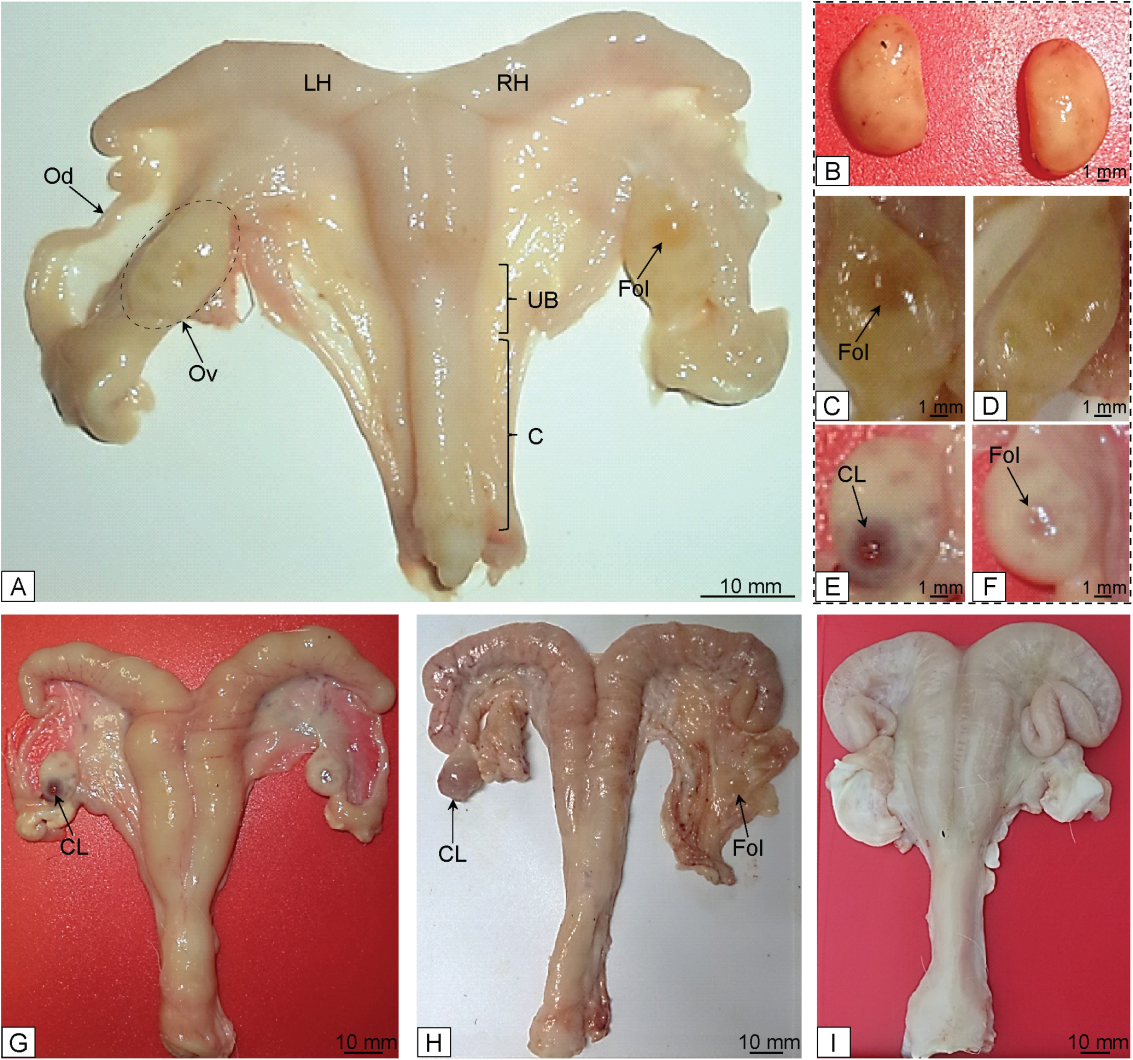
Characteristics of female white-tailed deer reproductive tract. (A) Fawns reproductive tract illustrating the ovary (Ov), oviduct (Od), left and right uterine horns (LH and RH, respectively), uterine body (UB), cervix (C), and large antral follicle (Fol); (B) ovaries of a young fawn with very low antral follicle activity; (C and D) ovaries of a fawn with the presence of mature antral follicle; (E and F) ovaries of a doe with presence of corpus luteum (CL) and Fol; and (G, H, and I) reproductive tract of does.

**Fig 2:**
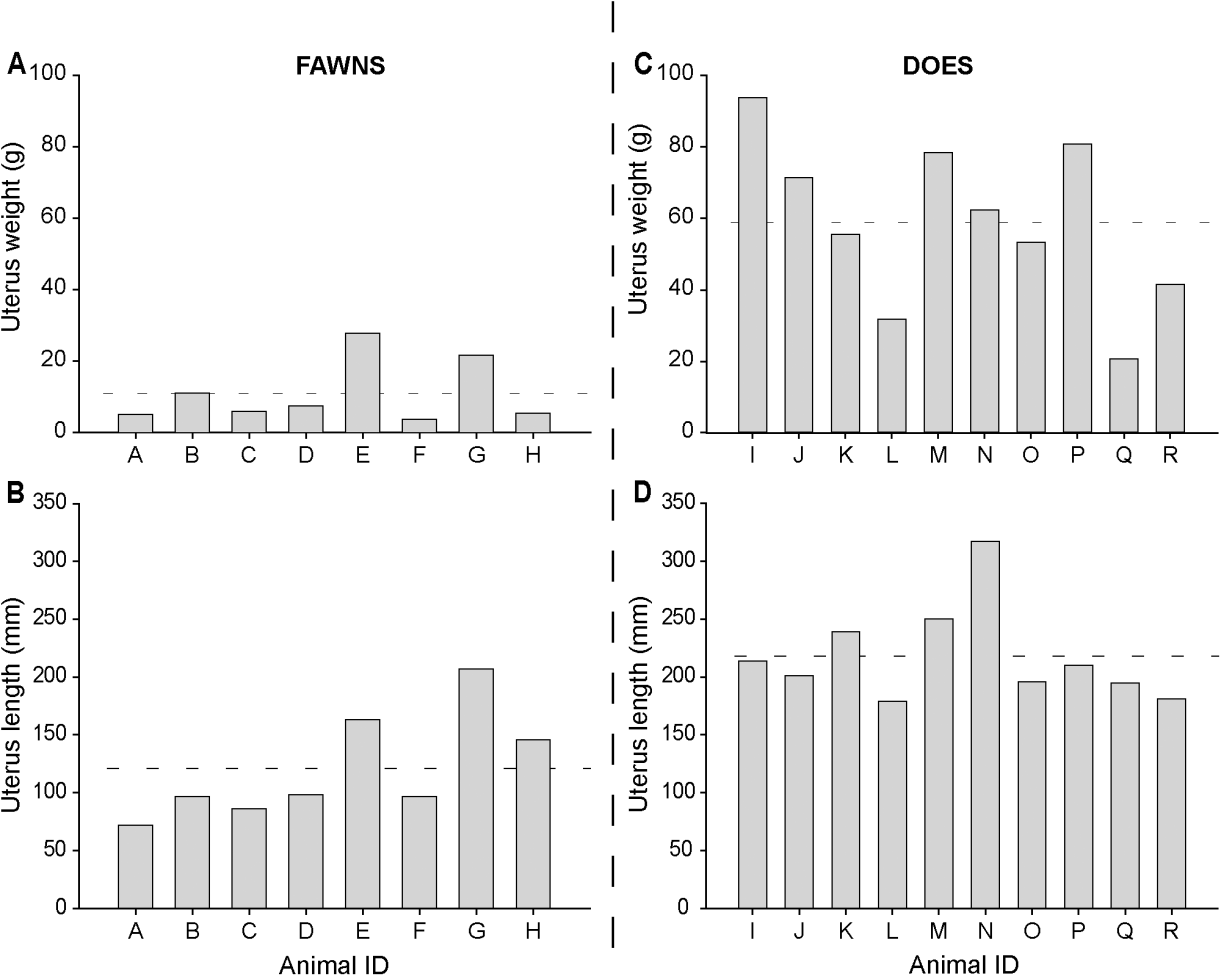
**Uterus weight (A and C) and length (B and D) in white-tailed deer fawns (A and B; n = 8) and does (C and D; n = 10).** Horizontal dashed line represents the overall mean within each figure.

Macroscopic features of the uterus and ovary of fawns and does are shown (**Fig 1; Table 1)**. The mean uterus weight was 37.7 ± 7.1 g (range, 3.8 to 93.7, CV = 80.2%) for all females. The uterus weight in does was approximately five times heavier (P < 0.001) than in fawns. Also, the whole length of the uterus had an overall mean of 175.0 ± 15.4 mm (range, 72 to 317, CV = 37.4%), and does had a uterus approximately two times larger (P < 0.002) than fawns. Uterus weight and length of each individual animal within age groups are shown (**Fig 2**). Likewise, the mean ovarian weight was 0.6 ± 0.05 g (range, 0.14 to 1.41, CV = 52%) among all females. Also, in does the ovary weight was two times heavier (P < 0.001) than in fawns. The mean ovary volume was 503.4 ± 46.8 mm^3^ (range, 146.6 to 1392.8, CV = 55.7%) for all females; however, the ovary volume in does was approximately 1.6 times larger (P < 0.05) than in fawns.

**Table 1:** Mean (± S.E.M.) values for macroscopic characteristics of the uterus and ovary in white-tailed deer fawns and does.

Macroscopic characteristics	Fawns	Does	P-value
	(n = 8)	(n = 10)	
**Uterus**			
Weight (g)	11.1 ± 3.2	59.0 ± 7.3	< 0.001
	(3.8–27.9)^*^	(20.8–93.7)	
Body length (mm)	38.3 ± 6.2	67.4 ± 3.1	< 0.001
	(20.0–63.0)	(58.0–90.0)	
Horn length (mm)^†^	41.3 ± 4.1	75.4 ± 4.3	< 0.001
	(24.0–87.0)	(55.0–125.0)	
Whole length (mm)^‡^	120.8 ± 16.4	218.2 ± 13.1	< 0.002
	(72.0–207.0)	(179.0–317.0)	
**Ovary**			
Weight (g)	0.4 ± 0.04	0.8 ± 0.07	< 0.001
	(0.1–0.7)	(0.3–1.4)	
Length (mm)	13.4 ± 0.5	15.5 ± 0.4	< 0.002
	(10.0–16.0)	(13.0–19.0)	
Height (mm)	8.9 ± 0.5	10.6 ± 0.5	< 0.02
	(7.0–14.0)	(6.0–14.0)	
Width (mm)	5.7 ± 0.2	6.7 ± 0.4	N.S.
	(4.0–7.0)	(4.0–11.0)	
Volume (mm^3^)	368.8 ± 33.1	611.1 ± 72.0	< 0.05
	(146.6–604.8)	(175.9–1392.8)	

*Range for macroscopic characteristics of the uterus and ovary.

^†^Left and right uterine horns combined.

^‡^Uterine body and horns combined.

N.S., non-significant.

**Fig 3:**
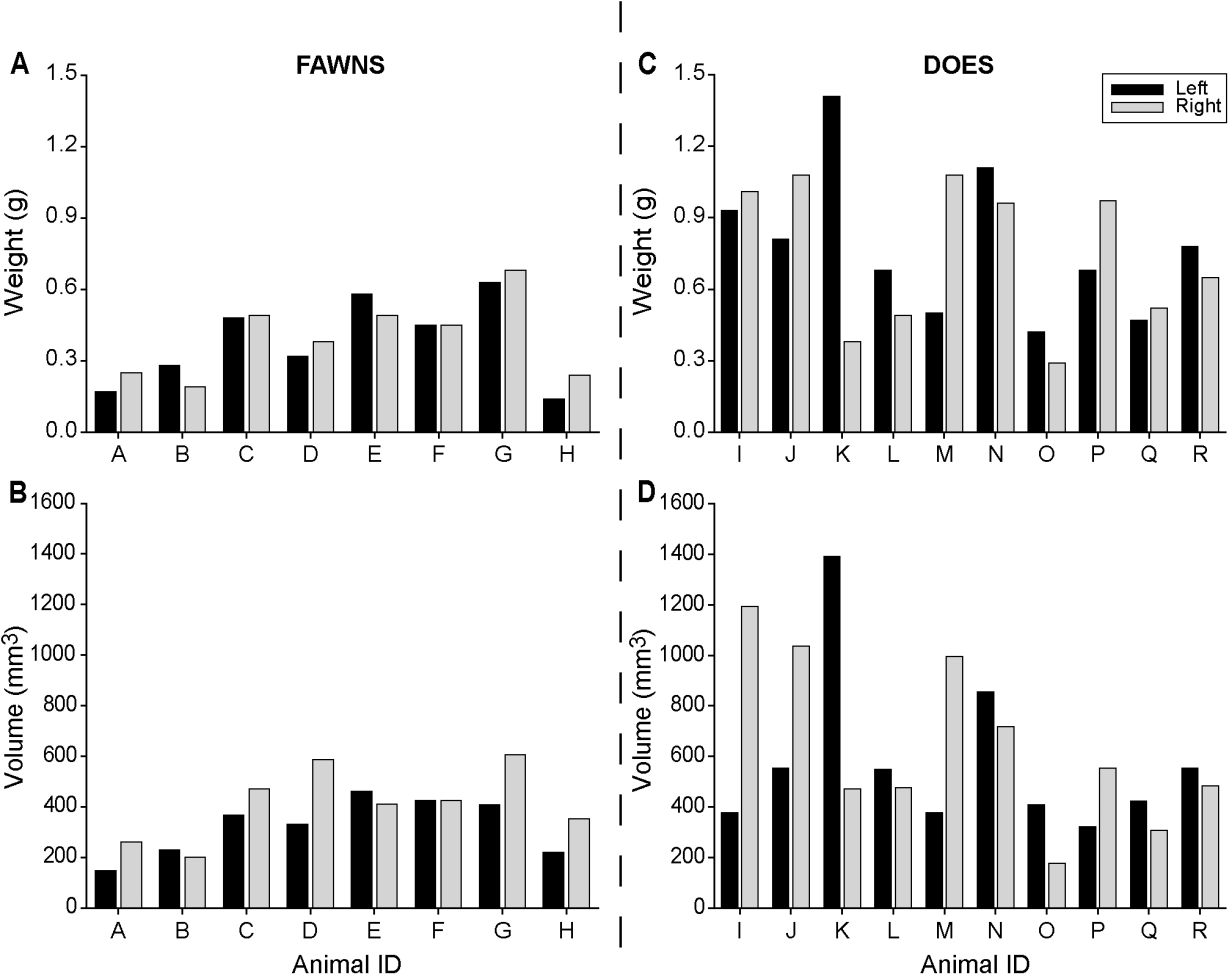
Ovary weight (A and C) and volume (B and D) for each side (left and right) in white-tailed deer fawns (A and B; n = 8) and does (C and D; n = 10).

Effect of side (left vs. right) was evaluated within and between fawns and does for uterus (horn length) and ovary (weight, length, height, width, and volume) features (**Table 2**). No difference (P > 0.05) between left and right side was observed for the end points evaluated for fawns and does. However, each uterus horn was longer (P < 0.05) and each ovary weight and volume greater (P < 0.05) in does, compared to fawns. The weight and volume of each left and right ovaries for all individuals are shown for fawns and does (**Fig 3**).

**Table 2:** Mean (± S.E.M.) values for macroscopic characteristics of the reproductive tract according to the side (left vs. right) of the uterine horn and ovary in white-tailed deer fawns and does.

Macroscopic characteristics	Fawns (n = 8)		Does (n = 10)	
	Left	Right	Left	Right
**Uterus**				
Horn length (mm)	044.0 ± 7.0^a^	038.5 ± 4.4^a^	073.5 ± 6.6^b^	077.3 ± 6.0^b^
**Ovary**				
Weight (g)	000.4 ± 0.1^a^	000.4 ± 0.1^a^	000.8 ± 0.1^b^	000.7 ± 0.1^b^
Length (mm)	012.9 ± 0.6^a^	013.9 ± 0.7^a^^b^	015.9 ± 0.5^b^	015.0 ± 0.6^a^^b^
Height (mm)	008.6 ± 0.4	009.3 ± 0.8	010.3 ± 0.6	010.9 ± 0.8
Width (mm)	005.4 ± 0.3	006.1 ± 0.3	006.4 ± 0.5	007.0 ± 0.7^†^
Volume (mm^3^)	323.5 ± 39.9^a^	414.1 ± 50.3^a^^b^	581.0 ± 102.3^b^	641.3 ± 106.0^b^

^a,b^ Within a row, values without a common superscript differed (P < 0.05).

^†^ Value tended (P < 0.08) to differ from left ovary in fawns.

### Presence of antral follicles, corpus luteum and albicans in ovaries of fawns and does

**Fig 4:**
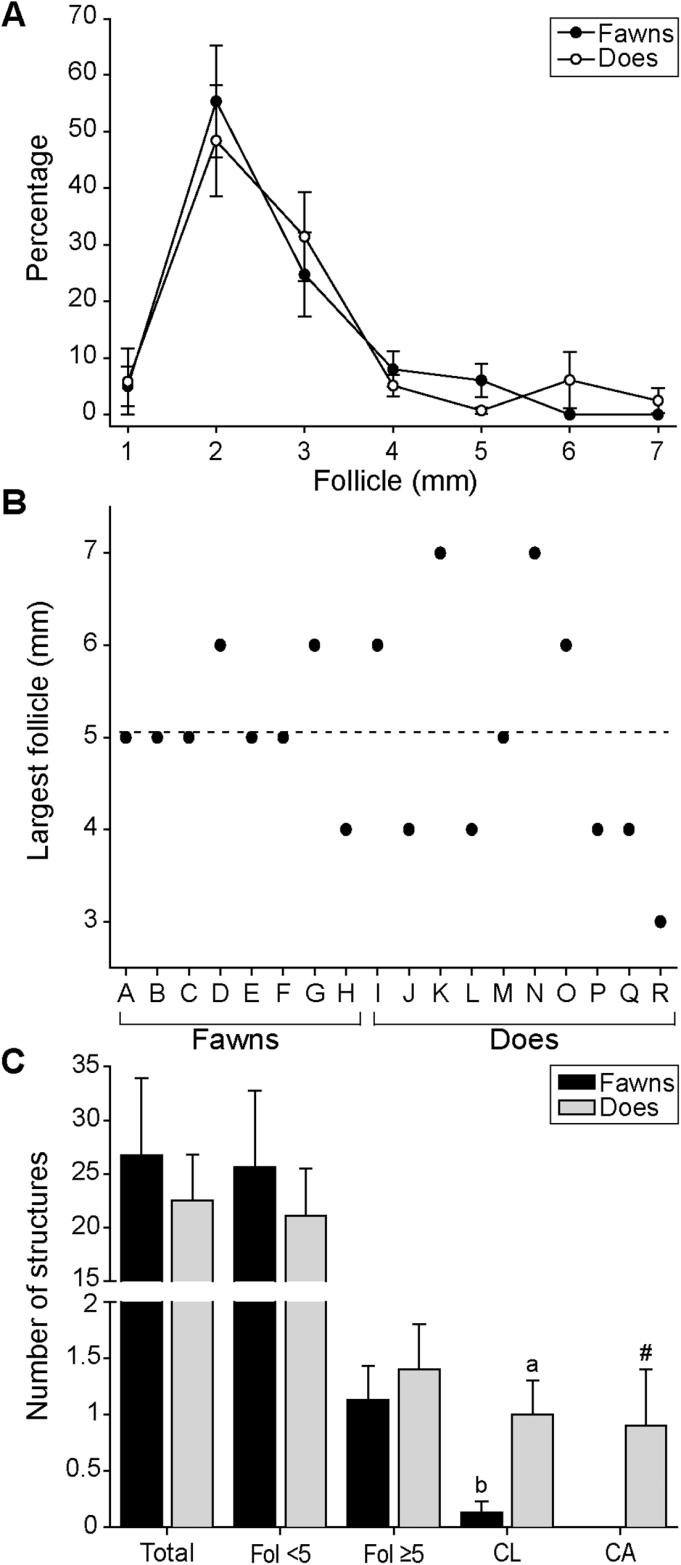
**(A) Mean (± S.E.M.) percentage distribution of antral follicles in the ovary of white-tailed deer fawns (n = 8) and does (n = 10); (B) Size of the largest follicle in each fawn and doe; (C) Overall mean (± S.E.M.) number of ovarian structures (total antral follicles; follicles <5 mm; follicles ≥5 mm; corpus luteum, CL; and corpus albicans, CA) in fawns and does.** ^a,b^ Values without a common superscript differed (P < 0.05) between fawns and does. ^#^ Value tended (P < 0.08) to differ between fawns and does.

The mean number of antral follicles was 24.4 ± 3.9 per ovary (range, 1 to 37, CV = 67.4%; data not shown) for all females; no difference (P > 0.05) was observed between fawns and does (26.8 ± 7.1 and 22.5 ± 4.3 follicles, respectively). In addition, no difference (P > 0.05) was observed for the number of antral follicles between left and right ovary within and between age groups (fawns, 12.4 ± 3.9 and 14.4 ± 3.3; does, 10.9 ± 2.3 and 11.6 ± 2.6). Moreover, the mean frequency of the follicle diameter (e.g., 1–7 mm) between left and right ovaries did not differ (P > 0.05) within and between fawns and does. In both age groups, the antral follicles with 2 mm in diameter represented 56% of all antral follicles (**Fig 4A**). The largest antral follicle was 5.1 ± 0.3 mm in diameter in all animals, and did not differ between fawns and does (5.1 ± 0.2 and 5.0 ± 0.4 mm, respectively). However, a greater variability in diameter of the largest follicle was observed in does than in fawns (**Fig 4B**). The total number of follicles, number of follicles <5 mm or ≥5 mm, and corpora lutea (CL) and corpus albicans (CA) are shown (**Fig 4C**). The number of follicles <5 or ≥5 mm did not differ (P > 0.05) between age groups. However, more (P < 0.05) follicles <5 mm (23.0 ± 3.9) were detected than follicles ≥5 mm (0.9 ± 0.2) in both age groups. The presence of CL (overall, 0.6 ± 0.2) and CA (0.5 ± 0.3) in the ovary was greater (P < 0.08–0.05) in does (60% and 60%) compared to fawns (12.5% and 0.0%).

### Influence of ovarian structures on weight and volume of ovaries in fawns and does

Strong positive correlation coefficients between ovary weight and volume within fawns (r = 0.80, P < 0.0001) and does (r = 0.81, P < 0.0001) were observed. Fawns had a moderately positive correlation (r = 0.55, P < 0.05) between area of ovarian structures and ovary volume. Does had moderately positive correlations between area of ovarian structures and weight (r = 0.46, P < 0.05) and volume (r = 0.52, P < 0.05).

### Preantral follicle size, population, and distribution

Diameter of preantral follicles and its oocytes (n = 1131; **Table 3**) were measured according to follicle classes, such as primordial (fawns, n = 155; does, n = 186), transition (fawns, n = 155; does, n = 187), primary (fawns, n = 155; does, n = 173), and secondary (fawns, n = 30; does, n = 90).

**Table 3:** Mean (± S.E.M.) diameter of preantral follicles and oocytes per classes in white-tailed deer fawns and does.

Age groups	Primordial		Transition		Primary		Secondary	
	(n = 341)		(n = 342)		(n = 328)		(n = 120)	
	Follicle	Oocyte	Follicle	Oocyte	Follicle	Oocyte	Follicle	Oocyte
**Fawns**	30.9 ± 0.3	17.4 ± 0.1	31.9 ± 0.2^a^	17.7 ± 0.1^†^	39.0 ± 0.1^a^	17.6 ± 0.2	206.3 ± 12.9	76.5 ± 3.6
	(22.3–38.6)^‡^	(14.3–19.8)	(26.3–39.2)	(15.4–20.4)	(34.8–42.6)	(13.6–21.7)	(83.5–338.8)	(45.4–115.1)
**Does**	31.4 ± 0.2	17.5 ± 0.1	31.2 ± 0.2^b^	17.4 ± 0.1	38.2 ± 0.2^b^	17.9 ± 0.2	222.5 ± 8.6	80.8 ± 2.3
	(26.6–36.5)	(15.0–19.9)	(25.7–36.8)	(15.1–19.8)	(26.6–42.6)	(13.1–28.6)	(86.1–359.7)	(47.7–114.4)
**Overall**	31.1 ± 0.1	17.5 ± 0.1	31.5 ± 0.1	17.5 ± 0.1	38.6 ± 0.1	17.8 ± 0.1	217.6 ± 7.2	79.5 ± 1.9
	(22.3–36.6)	(14.3–19.9)	(25.7–39.2)	(15.1–20.4)	(26.6–42.6)	(13.1–28.6)	(83.5–359.7)	(45.4–115.1)

^a,b^ Within the same column, values without a common superscript differed (P < 0.05).

^†^ Oocyte diameter in fawns tended to differ (P < 0.07) from does.

^‡^ Diameter range; diameter was measured on morphologically normal follicles/oocytes only.

**Fig 5:**
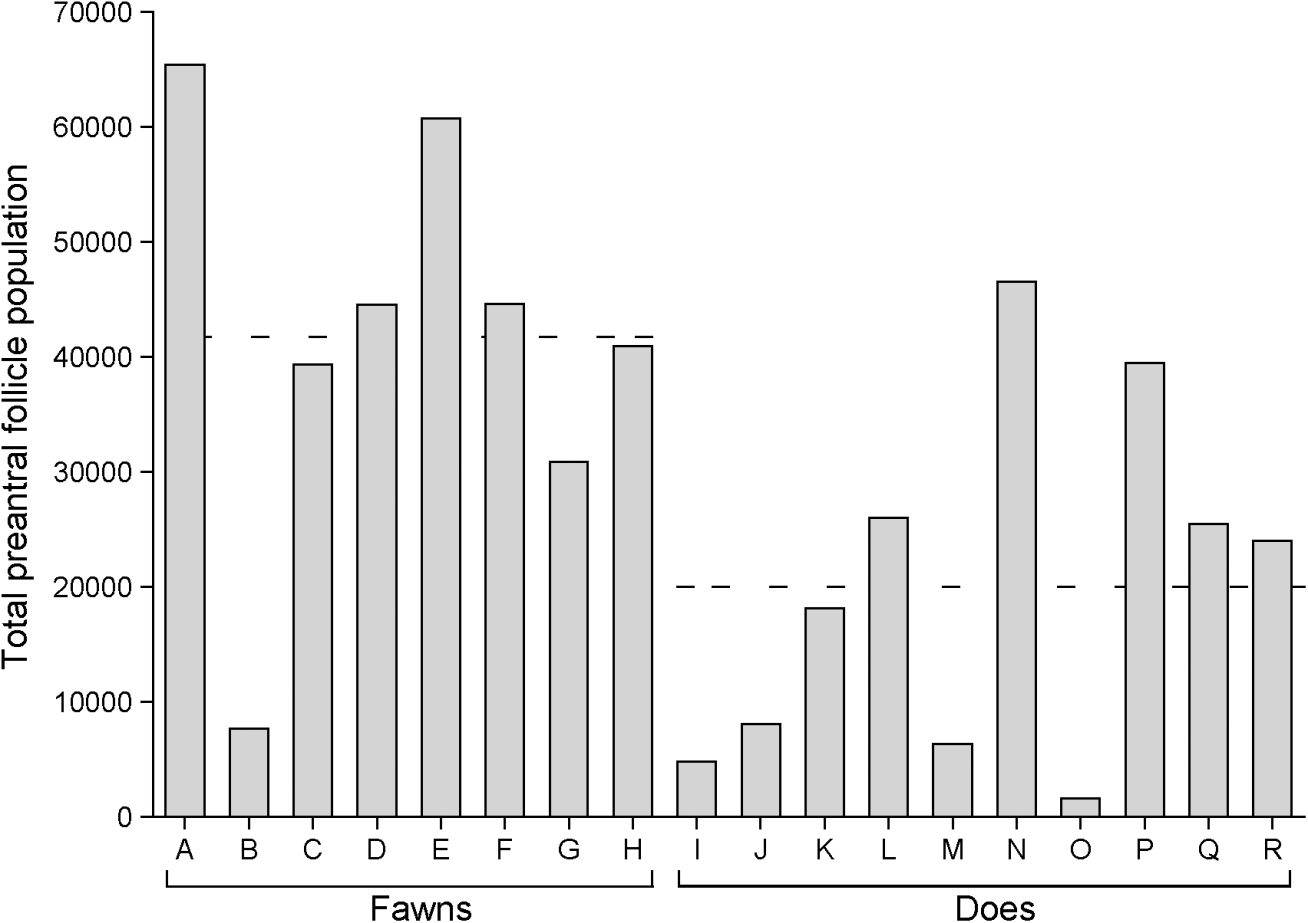
Preantral follicle population for each white-tailed deer fawns (n = 8) and does (n = 10). Dashed lines represent the mean for each age group.

**Fig 6:**
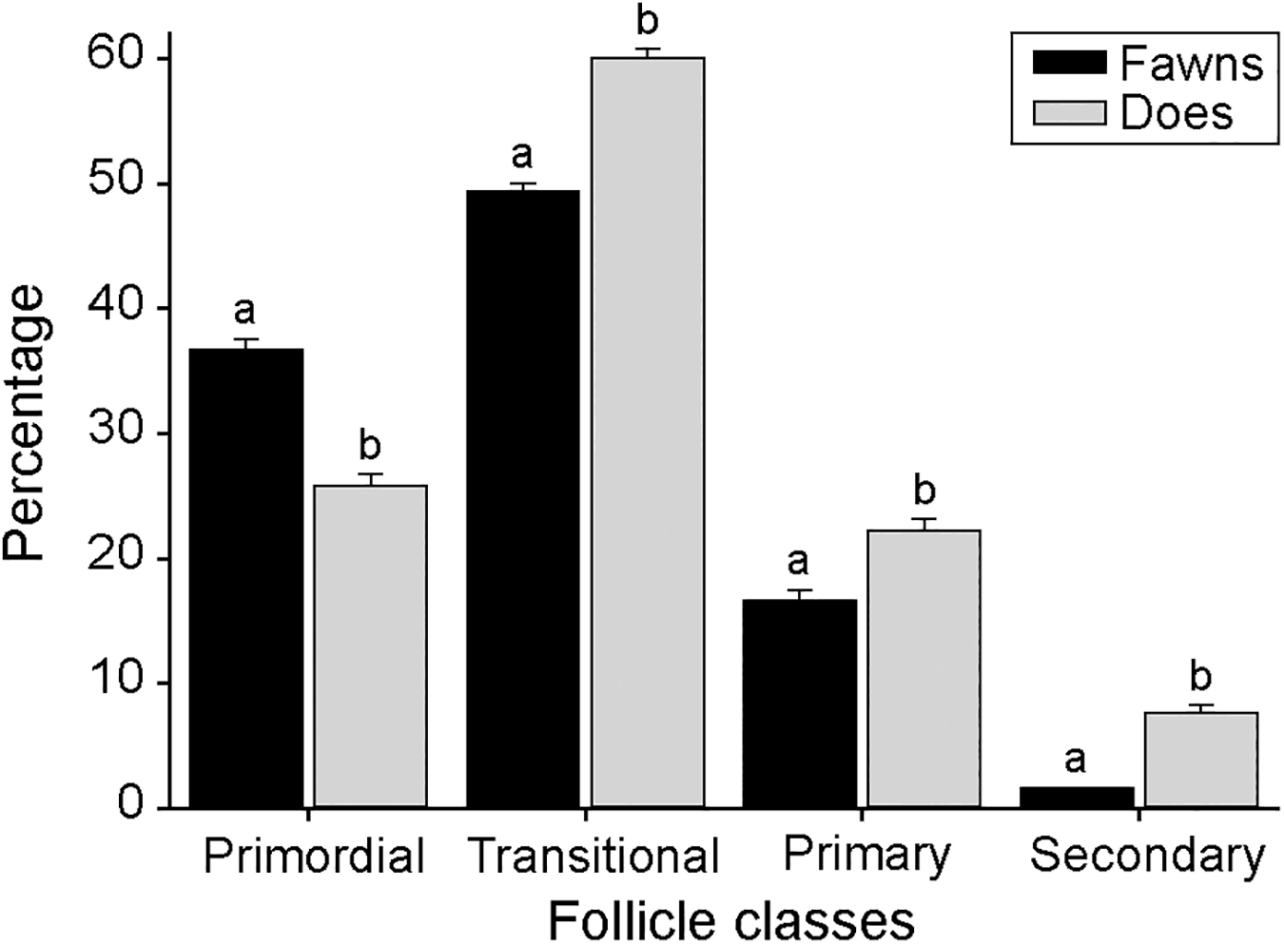
Preantral follicle distribution in the ovary of white-tailed deer fawns (n = 8) and does (n = 10). ^a,b^ Within follicle class, values without a common superscript differed (P < 0.05).

**Fig 7:**
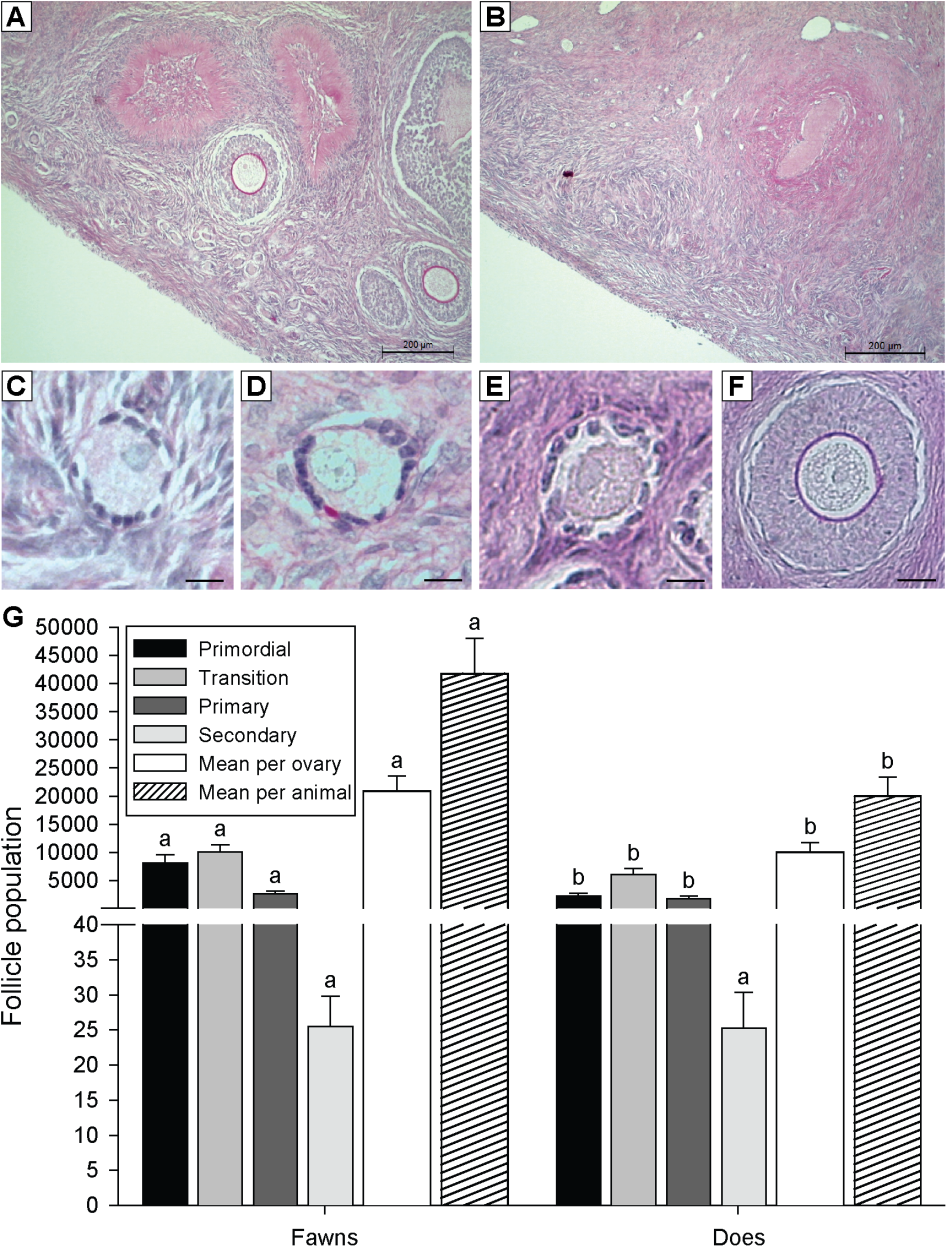
**Characteristics of preantral follicle population in the ovary of white-tailed deer (A) fawns and (B) does, and preantral follicles classes such as (C) primordial, (D) transition, (E) primary, and (F) secondary. (G) Preantral follicle population according to follicle class for white-tailed deer fawns (n = 8) and does (n = 10).** ^a,b^ Within the same types of columns, values without a common superscript differed (P < 0.05). Bars = 10 μm (**C–F**).

Preantral follicle population in ovaries of individual fawns and does is shown (**Fig 5**). Overall, white-tailed deer had an average of 14,839 ± 1,754 follicles per ovary. Fawns had more (P < 0.05) preantral follicles (41,754.4 ± 6,301.5) than does (20,018.6 ± 4,792.0). The percentage of follicle class distribution in ovaries of fawns and does is shown (**Fig 6**). Fawns had a greater (P < 0.05) percentage of primordial follicles than does. However, does had greater (P < 0.05) percentage of developing follicles (transition, primary, and secondary) compared to fawns. Except for secondary follicles, fawns had a greater (P < 0.05) population in all other follicle classes, compared to does (**Fig 7**).

**Fig 8:**
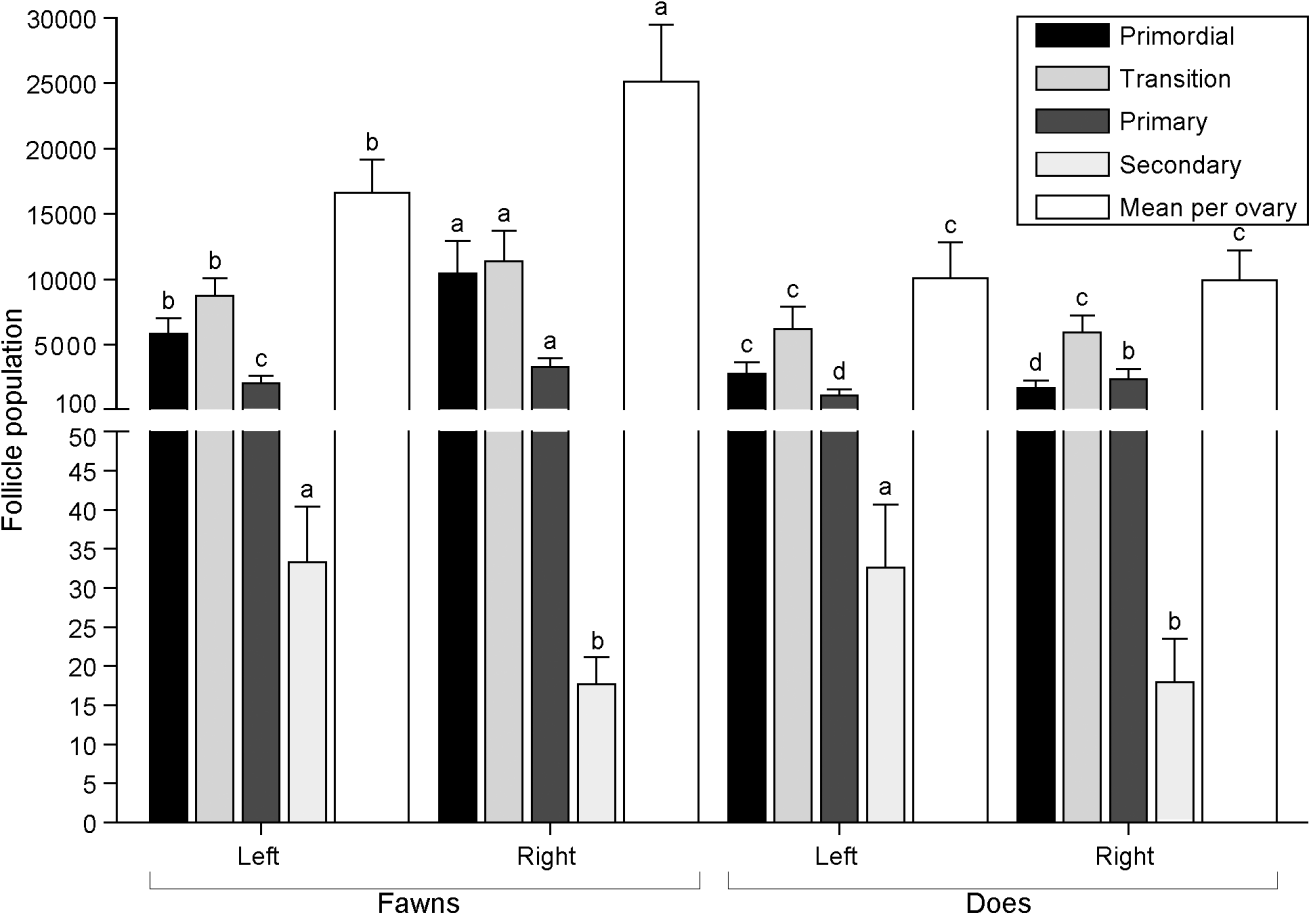
Mean (± S.E.M.) preantral follicle population, according to follicle classes, comparing left and right ovaries in white-tailed deer fawns (n = 8) and does (n = 10). ^a,b,c,d^ Within the same types of columns, values without a common superscript differed (P < 0.05) between left and right ovaries within each age group.

Follicle population was also recorded according to follicle class distribution within the left and right ovaries (**Fig 8**). In fawns, the right ovary had a greater (P < 0.05) total number of follicles, and number of primordial, transition, and primary follicles. In does, more (P < 0.05) primordial and primary follicles were seen in the left and right ovaries, respectively.

### Preantral follicle morphology

Overall, the total number of preantral follicles recorded in both ovaries of fawns and does was 60,394, where 51,598 follicles (85.4%) were classified as morphologically normal.

Regardless of follicle class and ovary side, fawns had a greater (P < 0.05) percentage of normal follicles than does (**Table 4**).

**Table 4:** Mean (± S.E.M.) percentage of normal preantral follicles in each follicular class according to age (fawns and does) and ovary side (left and right).

Follicle class	Fawns (n = 8)		Does (n = 10)		Mean ovaries	
					(left + right)	
	Left	Right	Left	Right	Fawns	Does
Primordial	88.1 ± 1.2^A^	84.9 ± 1.4^B^	77.4 ± 1.9^B^	78.3 ± 1.9^B^	86.5 ± 0.9 ^a^^,^^X^	77.8 ± 1.3^b^^,^^X^
	(n = 5215)^†^	(n = 9335)	(n = 3114)	(n = 1871)	(n = 14550)	(n = 4985)
Transition	86.6 ± 0.8^A^	88.5 ± 0.6^A^	77.9 ± 1.5^B^	80.9 ± 1.0^B^	87.5 ± 0.5^a^^,^^X^	79.4 ± 0.9^b^^,^^X^
	(n = 7921)	(n = 10344)	(n = 6905)	(n = 6609)	(n = 18265)	(n = 13514)
Primary	85.9 ± 1.9^A^	87.2 ± 1.5^A^	69.8 ± 22.8^B^	84.1 ± 1.7^A^	86.6 ± 1.2^a^^,^^X^	77.2 ± 1.7^b^^,^^X^
	(n = 1853)	(n = 3001)	(n = 1313)	(n = 2748)	(n = 4854)	(n = 4061)
Secondary	70.5 ± 7.3^A^	78.6 ± 8.8^A^	51.4 ± 6.9^B^	71.2 ± 9.1^A^^,^^B^	73.3 ± 5.6^a^^,^^Y^	58.3 ± 5.6^b^^,^^Y^
	(n = 47)	(n = 25)	(n = 60)	(n = 33)	(n = 72)	(n = 93)
Overall	87.6 ± 0.6^A^	88.2 ± 0.5^A^	77.0 ± 1.3^B^	81.9 ± 0.8^B^	87.9 ± 0.4^a^	79.4 ± 0.8^b^
	(n = 15036)	(n = 22705)	(n = 11392)	(n = 11261)	(n = 37741)	(n = 22653)

^a,b^ Within a row, values without a common superscript differed (P < 0.05) for mean ovaries (left + right).

^A,B^ Within a row, values without a common superscript differed (P < 0.05) among ovaries, between fawns and does.

^X,Y^ Within fawns and does, values of each follicle class without a common superscript differed (P < 0.05).

^†^ Total number of preantral follicles in each follicle class.

### Preantral follicle density and stromal cell density

Preantral follicle and stromal cell density in ovaries of fawns and does are shown (**Table 5**). Preantral follicle density ranged from 0 to 545 follicles/cm^2^ (CV = 103%) and differed within animals regardless of group. Furthermore, fawns had a greater (P < 0.05) follicle density compared to does. Stromal cell density in ovaries of fawns and does ranged from 36 to 90 cells/2,500μm^2^ (CV = 19%) and did not differ (P > 0.05) between age groups; however, difference (P < 0.05) was observed among animals within each age group.

**Table 5:** Mean (± S.E.M.) density of preantral follicles and stromal cells in ovaries of white-tailed deer fawns and does.

Animal ID	Age group	Preantral follicle density	Stromal cell density
A	Fawn	388.8 ± 14.6^k^	78.3 ± 0.6^i^
B	Fawn	041.8 ± 3.2^b^	68.6 ± 0.3^f^
C	Fawn	126.6 ± 7.8^g^^h^	75.9 ± 0.7^g^^i^
D	Fawn	160.3 ± 3.2^h^^i^	68.6 ± 0.3^f^
E	Fawn	175.4 ± 14.1^i^	59.6 ± 1.3^b^^c^
F	Fawn	111.3 ± 5.8^f^^g^	48.9 ± 1.5^a^
G	Fawn	085.2 ± 2.6^e^^f^	55.9 ± 0.3^b^
H	Fawn	218.4 ± 11.1^j^	48.4 ± 1.1^a^
I	Doe	013.6 ± 1.0^a^	72.3 ± 1.6^f^^g^^h^
J	Doe	014.9 ± 1.4^a^	63.1 ± 2.0^c^^d^^e^
K	Doe	074.8 ± 14.6^b^^d^	66.7 ± 0.7^d^^f^
L	Doe	066.8 ± 1.9^c^^d^^e^	77.3 ± 1.2^h^^i^
M	Doe	013.5 ± 0.8^a^	71.2 ± 0.5^f^^g^
N	Doe	087.5 ± 4.6^e^^f^	68.1 ± 0.9^e^^f^
O	Doe	005.8 ± 0.8^a^	63.4 ± 1.7^c^^d^^e^
P	Doe	082.3 ± 4.9^d^^f^	61.6 ± 0.9^c^^d^
Q	Doe	079.2 ± 7.5^c^^d^^e^	55.0 ± 1.3^b^
R	Doe	051.9 ± 3.0^b^^c^	47.7 ± 0.3^a^
Means	Fawns	156.4 ± 6.1^A^	63.1 ± 0.7^A^
	Does	049.0 ± 2.3^B^	64.6 ± 0.5^A^
Overall		095.8 ± 3.5	63.9 ± 0.4

^a–k^ Within a column, mean values of individual animals without a common superscript differed (P < 0.05).

^A,B^ Within a column, mean values for each age group (fawns and does) without a common superscript differed (P < 0.05).

### Effect of age on ovarian apoptosis

**Fig 9:**
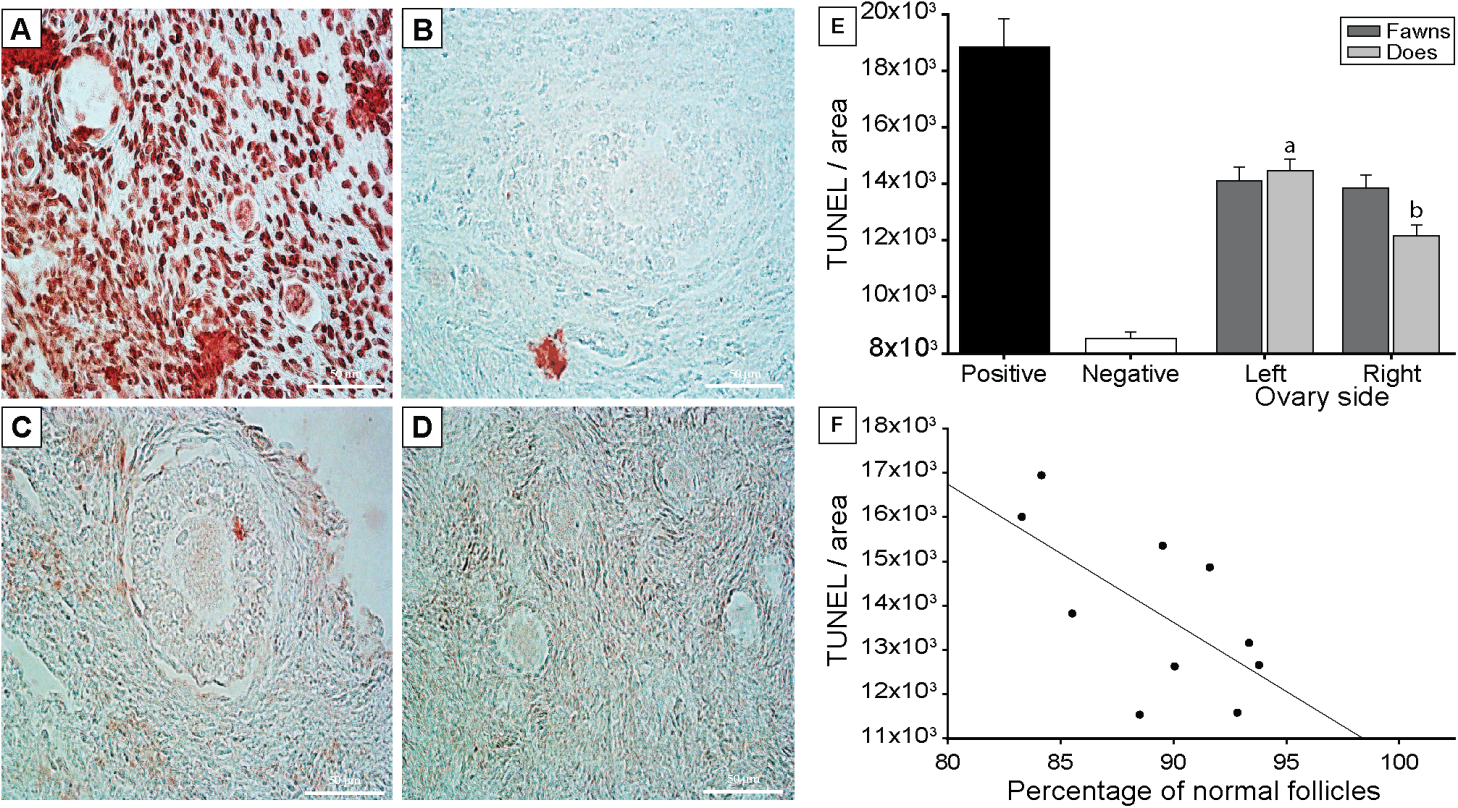
TUNEL quantification. **Representative images of apoptotic cells in white-tailed deer ovary.** (A) positive control, (B) negative control, (C) fawns, and (D) does. (E) Intensity of TUNEL staining per unit area for positive and negative controls, and left and right ovaries of fawns (n = 5) and does (n = 5). (F) Linear regression between the percentage of normal follicles and TUNEL quantification in fawns [TUNEL = 24640375.802 –(120834.742 * %Normal), r = –0.4, r^2^ = 0.16, P < 0.001]. Each dot represents one ovary (n = 10) of 5 fawns. ^a,b^ Within does, values without a common superscript differed (P < 0.05).

The quantification of apoptotic cells by TUNEL assay in ovaries of fawns and does is illustrated (**Fig 9A–9D**). Overall, no difference (P > 0.05) was observed between fawns and does. However, when evaluated the ovary side effect within each age group, fawns had a similar apoptotic signal in both ovaries, while does had greater (P < 0.05) apoptotic signal in the left than the right ovary (**Fig 9E**). Furthermore, in fawns, a negative effect of cell apoptosis on follicle morphology was confirmed by linear regression analyses (P < 0.001; **Fig 9F**). Also, in fawns, there was a negative correlation (r = –0.3, P < 0.001) between follicle density and TUNEL cells, and a positive correlation (r = 0.2, P < 0.05) between stromal cell density and TUNEL cells.

## Discussion

The importance of determining the ovarian reserve quantitatively and qualitatively has been emphasized in order to understand the variations that influence its establishment, and hence the reproductive lifespan of the individual and the impact on post-reproductive health **[26]**. The present study describes, for the first time, the preantral follicle reserve including preantral follicle population, density, morphology, and distribution of classes in the ovary of white-tailed deer within two different age groups (fawns and does). In addition, we have demonstrated that apoptosis in the ovarian tissue leads to a negative effect on preantral follicle morphology. Furthermore, we have described, in a more comprehensive fashion, the uterus and ovary dimensions of white-tailed deer. The present study confirms as previously demonstrated in other species **[21–23]** that ageing causes a drastically negative effect on the preantral follicle population, morphology, and density. Therefore, the results found in this study contribute to: (1) nourish the knowledge of reproductive physiology in the white-tailed deer; (2) develop protocols for preservation of germ cells of threatened species; (3) sustain the development of better contraceptive treatments for fertility control of Cervidae species; and (4) evaluate the white-tailed deer as a potential animal model for reproductive studies in women.

In the present study, the preantral follicle population estimated in white-tailed deer ovaries (14,839 follicles per ovary) was considerably lower than most of other mammalian species, such as goats and sheep ≈38,000 **[14, 41]**; cattle, Bos indicus ≈40,000, Bos taurus ≈90,000 **[16]**; swine, ≈210,000 primordial follicle **[42]**; wild mammals, collared peccaries ≈33,000 **[43]**, elephants ≈240,000 **[18]**, and rhesus monkey, range, 23,000 to 226,000 **[44]**; and women ≈89,000 **[19, 45]**. However, in mice because of the small ovary size, the preantral follicle population (e.g., 2000 follicles per ovary, **[46]**) is smaller when compared with other species. Variations among and within species regarding to the number of preantral follicles in the ovary have been related to the following factors: breed, body weight, age, ovulation rate, and reproductive phase **[16, 23, 42, 43]**. Despite all different factors that can affect preantral follicle population, our study clearly demonstrated that the follicle reserve is sharply reduced as white-tailed deer age. Preantral follicle density reported in this study (95.8 follicles per cm^2^) is representing the follicle density per area of the entire (cortex and medulla) ovary. This information may differ from other studies (0–109 preantral follicles/mm^2^, **[47]**; ≈53.4, **[48]**; ≈15.0, **[13])** where only the ovarian cortex was considered to determine the preantral follicle density per mm^2^. However, when the medulla is not considered, there is an underestimation in the ovarian preantral follicle density. This affirmation is corroborated by a study **[49]** performed in human ovarian medulla tissue, which demonstrated that follicle density varied from 0 to 9,824 follicles per gram of medulla. Furthermore, the structures (antral follicles and corpora lutea) present in the ovary directly affect the preantral follicle density and its distribution **[15]**. In addition, ovarian volume has been demonstrated as an important factor to be used in models to study the spatial distribution of follicle reserve and activation of follicular growth in the mammalian ovary **[22]**. Therefore, the entire ovarian size, not solely the cortex, should be taken into account to determine the follicle reserve, where a ratio of a total number of preantral follicles per ovarian area or volume for each species may be a better parameter for inter-species comparisons.

Independent of preantral follicle classes, fawns had more primordial follicles than does. Therefore, it seems that an increase in primordial follicle activation occurs when fawns achieve puberty and start being reproductively active. In fawns and does, the transition follicle was the most predominant class in both ovaries. Follicle development in the animal’s ovary is highly affected by age and the reproductive phase **[15, 22]**. In addition, the number of primordial follicles has been demonstrated to decline in an almost linear fashion curve according to age in mares **[23]** and in women **[21, 50, 51]**. Besides the reduction of preantral follicle population in does, preantral follicle morphology was also more negatively affected in does than in fawns for all preantral follicle classes. These findings are in agreement with previous reports in mares **[23]** and women **[21]**, which reported higher incidence of healthy primordial follicles in ovaries of young females and higher incidence of follicle atresia with age.

The death of germ cells of the ovarian reserve occurs mainly by autophagy or apoptosis **[26]**. This study has demonstrated that apparently apoptosis in the ovarian tissue does not differ between does and fawns. However, the apoptosis of ovarian tissue had a negative effect on preantral follicle morphology. Apoptosis on preantral follicle cells has been largely described in several species **[24, 25]**. Previous studies have demonstrated apoptosis specifically on preantral follicles cells (e.g., oocyte, and granulosa), overlooking that preantral follicles are enclosed in the ovarian tissue, which contains a large population of different types of cells. It has been shown that preantral follicles are highly dependent on the surrounding cells for survival and development **[52, 53]**. Therefore, our study reinforces the assumption that preantral follicles are dependent and very sensitive to variations of the surrounding ovarian stromal cells.

To the best of our knowledge, this is the first study to describe in detail the macroscopic morphological measurements of the ovary and uterus of white-tailed deer fawns and does. The ovary, and uterus size and weight, according to the age, has followed a similar development pattern as observed in other mammalian species **[54, 55]**. In addition, our exhaustive description of the white-tailed deer reproductive tract has reinforced the effect of age on ovary and uterus development.

In conclusion, this study shows, for the first time, the preantral follicle population, rate of morphologically normal follicles, distribution by classes, density of preantral follicles and stromal cells, dimensions and weight of uteri and ovaries, and quantification of antral follicles, corpora lutea and albicans in white-tailed deer fawns and does. Moreover, the effect of age on the ovarian reserve of white-tailed deer was quantitatively and qualitatively supported. A complete understanding of the white-tailed deer ovarian biology will provide more insights for developing new methods of fertility control.
